# Machine Learning–Based First-Trimester Antenatal Risk Prediction for Adverse Maternal and Neonatal Outcomes: Multicenter Model Development Study

**DOI:** 10.2196/88450

**Published:** 2026-08-26

**Authors:** Sarah Li, David Y Y Tan, Jingxian Zhang, Aniza P Mahyuddin, Harshaana Ramlal, Sebastian E Illanes, Max Monckeberg, Alejandra F Plaza, Maria L Paz Morgan, Matthew W Kemp, Kee Yuan Ngiam, Peter Lindgren, Marius Kublickas, Karolina Kublickiene, Ruifen Weng, Sidney Yee, Mahesh Choolani

**Affiliations:** 1 Department of Obstetrics and Gynaecology, National University Hospital, Singapore, Singapore, Singapore; 2 Diagnostics Development Hub, Agency for Science, Technology and Research, Singapore, Singapore; 3 Department of Obstetrics and Gynaecology, NUS Yong Loo Lin School of Medicine, National University of Singapore, 1E Kent Ridge Road, NUHS Tower Block, Level 12, Singapore, Singapore, 119228, Singapore, 65 93889316; 4 Department of Obstetrics and Gynecology and Reproductive Biology Laboratory, Universidad de los Andes, Santiago, Chile; 5 IMPACT, Center of Interventional Medicine for Precision and Advanced Cellular Therapy, Santiago, Chile; 6 Women and Infants Research Foundation, King Edward Memorial Hospital, Perth, Australia; 7 Department of Surgery, National University Hospital, Singapore, Singapore; 8 Department of Women’s and Children’s Health, Karolinska Institutet, Stockholm, Sweden; 9 Center for Fetal Medicine, Karolinska University Hospital, Stockholm, Sweden; 10 Department of Clinical Science, Intervention and Technology, Renal Medicine unit, Karolinska Institutet, Stockholm, Sweden; 11 Innovation and Enterprise, Agency for Science, Technology and Research, Singapore, Singapore

**Keywords:** machine learning, obstetrics and gynecology, antenatal care, risk predictions, antenatal risk stratification, adverse pregnancy outcomes

## Abstract

**Background:**

Maternal outcomes remain inequitable worldwide. Severe morbidity persists, and current risk assessment tools are largely arbitrary, focusing on biomedical factors while overlooking social determinants of health. There is a need for data-driven AI models to improve early pregnancy risk identification and management.

**Objective:**

The study aimed to develop and internally validate first-trimester AI-based antenatal risk assessment models across three geographically and socioethnically diverse populations (Sweden, Chile, and Singapore) and to compare their performance with existing clinical risk assessment strategies.

**Methods:**

We conducted a retrospective population-based study using routinely collected first-trimester data from over 700,000 pregnancies from Sweden, Chile, and Singapore. Separate machine learning models predicting a composite of adverse maternal and neonatal outcomes were trained and internally validated for each population. Input variables were limited to information available at or before 14 weeks’ gestation. Model discrimination, measured by the area under the receiver operating characteristic (AUROC) curve, was compared with corresponding proxies for real-world first-trimester risk assessment approaches in each setting. Model interpretability was assessed using Shapley additive explanations.

**Results:**

The prevalence of the composite adverse outcome was 10.40% (75,647/727,354) in Sweden, 21.94% (1302/5934) in Chile, and 16.25% (6145/37,813) in Singapore. In Sweden, the guideline-based risk assessment achieved an AUROC of 0.53, compared with 0.65 for the LightGBM (light gradient boosting machine) model (*P*<.001). In Chile, the midwifery-led risk assessment achieved an AUROC of 0.52, versus 0.65 from the traditional machine learning LightGBM model (*P*<.001). In Singapore, the health care professional–based risk assessment reached an AUROC of 0.56, compared with 0.60 for the LightGBM model (*P*<.05). In the Swedish and Singapore cohorts, sociodemographic variables were among the most influential predictive features. At a false-positive rate of 50.0%, the sensitivities for predicting the primary composite adverse outcome were 69.17%, 69.62%, and 64.93% for the Sweden, Chile, and Singapore models, respectively. At a 30.0% false-positive rate operating point, the sensitivities were lower, at 50.38%, 55.38%, and 44.75%, respectively, but with higher positive predictive values of 16.31%, 34.53%, and 22.32%, respectively. The model calibration plots showed reasonable agreement in Sweden and Singapore, whereas the Chile model showed poorer calibration, with a calibration slope of approximately 1.45, indicating underconfidence.

**Conclusions:**

AI-based models developed using first-trimester data generally demonstrated improved performance compared with existing first-trimester clinical risk stratification strategies across three distinct populations. These findings suggest the potential feasibility of population-specific, AI-enabled risk stratification as a clinical decision support tool and highlight the potential value of integrating social, demographic, and behavioral determinants into antenatal risk assessment frameworks to support more equitable and personalized antenatal care.

## Introduction

Ensuring a safe and healthy pregnancy remains a cornerstone of global health, carrying profound implications for the well-being of women, children, and broader society. Despite advances in medical care, significant disparities in maternal outcomes persist, preventable complications continue to pose challenges across diverse health care systems, and maternal mortality remains a critical public health concern [1,2]. However, severe maternal morbidity (SMM) is arguably an equally, if not more, important indicator of the quality of maternity care, particularly in countries with relatively low maternal mortality ratios or small geographical populations [3]. For every maternal death, many more women experience serious, life-threatening conditions related to pregnancy and childbirth, with major obstetric hemorrhage and severe hypertensive disorders being the leading causes. Alarmingly, between 2000 and 2020, the maternal mortality ratio stagnated in 133 countries and increased substantially in 17, while SMM complicated up to 8% of hospital deliveries [4,5]. Reviews of maternal deaths and SMM cases consistently indicate that the majority are potentially preventable. Failure to identify cases as high risk, delays in diagnosis, and subsequent delays in treatment each contributed to the maternal deaths [6-8]. These concerning trends underscore the urgent need for improved strategies in identifying at-risk pregnancies before complications arise.

Pregnancy risk assessment remains a cornerstone of maternity care. It enables the early identification of pregnancies that may benefit from targeted interventions—either to prevent or delay adverse outcomes, or to facilitate timely intervention or management of complications [9,10]. Nevertheless, in many maternity care settings, the current risk assessment systems rely on arbitrary criteria, are imprecise, and are often dependent on human experience [11]. Furthermore, traditional systems often exclude sociocultural determinants of health, such as socioeconomic status, education, and ethnicity, largely due to the challenges in quantifying and integrating their impact into clinical risk models [12,13].

Given these limitations, there is an increasing move toward data-driven approaches capable of capturing the breadth and complexity of maternal health risks. AI technology, particularly ML, offers a promising avenue in this evolution, allowing the incorporation of multimodal data, including nontraditional and nonclinical data, into health care, aligning with the broader goal of universal, equitable, and holistic medical care [14,15]. These technologies can identify complex patterns within large datasets that incorporate both clinical and sociodemographic data, enabling more accurate and individualized categorization of pregnancy risk. Despite the growing interest in these technologies for predicting pregnancy complications, a significant research gap remains: most existing models are limited to specific obstetric outcomes, developed in homogeneous populations, and lack rigorous validation across diverse populations [16-18]. Crucially, no AI-based antenatal risk assessment model has been rigorously evaluated against standard practice in randomized clinical trials, nor is any model yet ready for integration into routine obstetric care [19].

To address this gap, we aimed to develop and internally validate first-trimester AI-based antenatal risk assessment models across 3 geographically and socioethnically distinct populations (Sweden, Chile, and Singapore) and to test whether AI-based models more accurately classify pregnancy risk than current antenatal practice.

## Methods

### Study Design and Datasets

**Figure 1.**
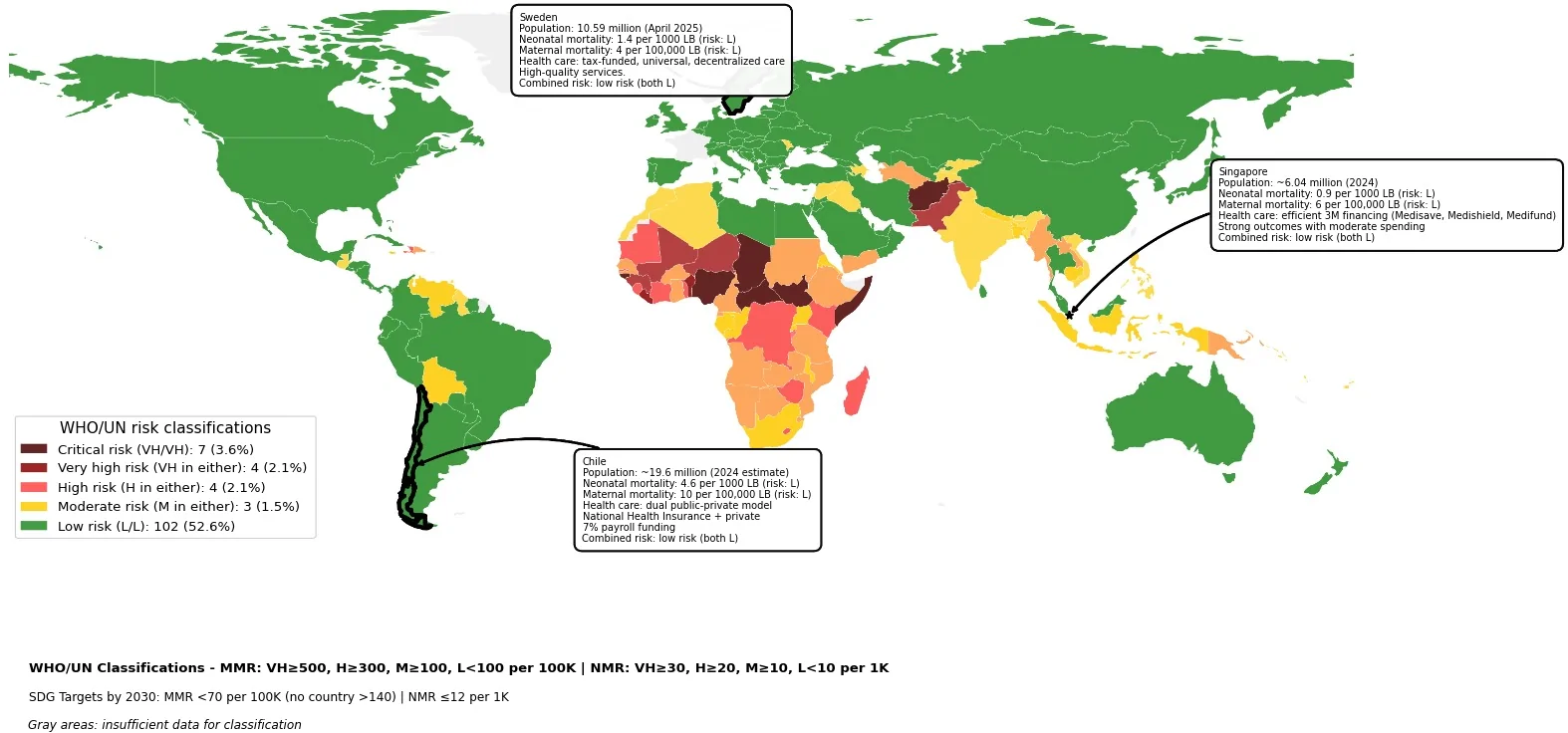
Global risk of maternal and neonatal mortality according to 2023 World Health Organization and United Nations Children's Fund estimates. H: high; L: low; LB: live births; M: moderate; MMR: maternal mortality ratio; NMR: neonatal mortality rate; SDG: Sustainable Development Goal; UN: United Nations; VH: very high; WHO: World Health Organization.

We retrospectively developed ML models using routinely collected historical data from Sweden, Chile, and Singapore. For Sweden, data were derived from the Swedish Pregnancy Register [20], linked with sociodemographic data from Statistics Sweden [21]. Pregnancies and deliveries between 2014 and 2021 were included. For Chile, data were obtained from Hospital Parroquial de San Bernardo, an intermediate-complexity maternity unit in Santiago that provides care for high-risk pregnancies. Pregnancies and deliveries between January 2018 and July 2022 were included. The Singapore data were extracted from a single tertiary maternity center, the National University Hospital, for deliveries between 2015 and 2022. Figure 1 depicts a heatmap of global maternal and neonatal mortality risk based on World Health Organization and United Nations Children’s Fund 2023 estimates, with detailed data for each country included in the analysis.

### Ethical Considerations

This study involved secondary analysis of routinely collected clinical and registry data and was reviewed and approved by the relevant institutional review boards at all 3 participating centers: National University Hospital (Singapore; DSRB 2021/00760), Karolinska University Hospital (Uppsala, Sweden; Dnr: 2020‐04437), and Hospital Parroquial de San Bernardo (Santiago de Chile; Register: 17072022). The requirement for individual informed consent was waived by all approving ethics committees. All datasets were anonymized or deidentified prior to analysis. No directly identifiable personal information was accessible to the study team. Data were stored in accordance with institutional data governance policies. No financial compensation was provided to participants. No images, figures, or supplementary materials in this manuscript include identifiable information about individual participants.

### Model Input Features

Input features were selected if they could be ascertained in the first trimester (before 14 weeks’ gestation) and classified into these domains: maternal sociodemographic characteristics, maternal past medical history, current obstetric characteristics, and laboratory investigations. Investigators in each country independently determined which features met these criteria and were applicable within their health care context as part of an ML model for antenatal risk assessment. The final feature sets included 33 variables for Sweden, 36 for Chile, and 20 for Singapore.

### Outcomes

The primary study outcome was a composite of severe adverse maternal, fetal, or neonatal events, including mortality, designed to capture overall pregnancy health rather than isolated obstetric complications. Neonatal morbidity indicators were selected for their substantial contribution to health care burden and included gestational age at delivery <37 weeks, birth weight <2500 g, stillbirth, 5-minute Apgar score <4, and the need for neonatal intensive care unit, including mechanical ventilation and hypoxic-ischemic encephalopathy. Maternal morbidity indicators were based on internationally recognized components of SMM and included delivery blood loss >1500 mL, eclampsia, placental abruption, embolism, uterine rupture, and admission to critical care (surgical high dependency or intensive care unit) for any cause. Maternal mortality indicators included death from any obstetric cause occurring more than 42 days but less than 1 year after delivery. Outcomes were harmonized across the 3 datasets, with the exception of maternal admission to critical care, which was not available in the Sweden dataset. A sensitivity analysis was performed in which admission to critical care was removed from datasets in which its percentage contribution to the primary composite exceeded 2%.

### ML Preprocessing, Model Training and Fine-Tuning, Ensembling, and Comparison

In this study, data from each country were randomly partitioned into training (80%) and testing (20%) sets using a fixed random seed of 42, with stratification by outcome label. Variables that were unavailable in certain populations were not imputed across countries but were simply not included in those country-specific models. Within each country’s dataset, missing values for available variables were handled using mean (continuous) or mode (categorical) imputation, with parameters derived exclusively from the training partition and subsequently applied to the corresponding test set to avoid data leakage. This was to ensure a consistent and transparent preprocessing approach across datasets. While this approach may attenuate variance, it is unlikely to materially affect comparative model performance. Gaussian noise was injected into continuous features in the training datasets to reduce overfitting and improve generalizability. To prevent data leakage, all preprocessing statistics were computed exclusively from the training partition before being applied to the test set. A sensitivity analysis was performed to assess the potential confounding by the COVID-19 pandemic on the Singapore dataset since its data collection period overlapped with the pandemic years (2020 to 2022). For the Sweden dataset, only a small proportion of records were from the start of the COVID-19 pandemic. For the Chile dataset, this was not possible due to the much smaller sample size. Tabular Prior-Data Fitted Network (TabPFN; version 2.1.0) and traditional ensemble methods in AutoGluon (version 1.4.0) were used for model building. TabPFN is a transformer-based architecture pretrained on millions of synthetic datasets and designed for tabular data classification [22].

TabPFN was further fine-tuned using the Sweden dataset (largest size) with batches of 15,000 samples using a learning rate of 1e-5 and a batch size of 5. The AdamW optimizer and ReduceLROnPlateau scheduler were used to enhance stability. Training proceeded for up to 10,000 iterations, with adaptive early stopping applied when no further improvement in validation loss was observed or when the time limit was reached. Model checkpoints were saved and reloaded to continue training within graphics processing unit constraints. In the Singapore dataset, the same fine-tuning was performed using mini-batches of 10,000 samples, and the AdamWScheduleFree optimizer. For the smaller Chile dataset, fine-tuning was performed using the default AdamWScheduleFree optimizer with the same learning rate and batch size. The dataset was processed in a single pass.

Additional experiments explored transfer learning by initializing training with the Sweden-fine-tuned model. To enrich predictive performance, embeddings from TabPFN were extracted and concatenated with raw features. These concatenated features were used as input for AutoGluon’s TabularPredictor. AutoGluon trained and ensembled multiple algorithms, including LightGBM (light gradient boosting machine), CatBoost, XGBoost, Extra Trees, Random Forest, and K-Nearest Neighbors, dynamically applying 8-fold bagging and 2-level stacking. Comparison of TabPFN against well-established gradient boosting and tree-based methods was obtained from ensembled algorithms. Label class imbalance was mitigated using TabPFN’s built-in probability balancing. The TabPFN embedding pipeline was used for exploratory benchmarking only and was not part of the final selected traditional models reported in the primary results. Models were evaluated on an independent test set for each country using the area under the receiver operating characteristic (AUROC) curve. For each dataset, the best-performing model was selected for comparison with standard antenatal risk assessment for categorizing high-risk and low-risk pregnancies. Model calibration was assessed using calibration plots comparing predicted probabilities against observed outcome frequencies across deciles of predicted risk, with calibration-in-the-large and calibration slope reported. In addition, sensitivity–positive predictive value (PPV) trade-offs at different clinically plausible thresholds were calculated. Lastly, we trained separate models for neonatal-only and maternal-only outcomes and compared the AUROC across outcome types and countries.

### Comparison With Standard Antenatal Risk Assessment in Each Population

To contextualize model performance, the best-performing ML model from the steps above was compared against proxies for the existing first-trimester antenatal risk assessment approach used within each population.

In Sweden, first-trimester risk categorization data were not directly available from the national registers. Accordingly, we retrospectively applied the clinical criteria currently used by Swedish antenatal care professionals to define a “high-risk pregnancy” to serve as the reference standard.

In Chile, antenatal risk assessment is typically initiated by community midwives during the first antenatal contact. Pregnant women are considered “at risk” if they meet predefined criteria across mental health, social, medical, or obstetrical domains. However, referral to a high-risk antenatal care pathway ultimately depends on the clinician’s discretion. In the Chilean dataset, information regarding first-trimester referrals to high-risk pregnancy clinics was available and used as a proxy for clinical risk categorization.

In Singapore, information on first-trimester referral to high-risk pregnancy services was not available in the retrospective dataset. Instead, this information was obtained from a separate prospective birth cohort capturing detailed antenatal and delivery data, with referral to high-risk services used as a surrogate for health care professional–based risk assessment.

For each country, AUROC was calculated to evaluate the performance of the real-world risk assessment in identifying pregnancies with the composite adverse outcome, enabling direct comparison with the corresponding AI-based model.

### Statistical Methods

All analyses were performed using Python SciPy-Stats (version 1.15.3) and the R pROC package (version 1.16.2). *P* values were calculated with the chi-square test for categorical variables and the Kruskal-Wallis test for continuous variables between the three groups. The prevalence of the composite primary outcome, as well as the prevalence of the individual components, was tabulated for each population dataset. As mentioned above, the ML models’ performance was evaluated using the AUROC curve and the corresponding 95% CI. Model calibration was reported using calibration plots. In addition, sensitivity, specificity, PPV, and negative predictive value were reported at false-positive rates (FPRs) of 50.0% and 30.0%. Comparative performance of ML models versus existing antenatal risk categorization approaches was assessed using the DeLong test for paired (Sweden and Chile) and unpaired (Singapore) AUROC, with statistical significance set at *P*<.05. Decision curve analysis (DCA) was conducted to assess the net clinical benefit of the ML models across clinically relevant threshold probabilities, in comparison with “screen all,” “screen none,” and the current clinical risk assessment strategies. Feature importance for the best-performing ML model in each dataset was evaluated using Shapley additive explanations (SHAP) analysis to identify the top 10 features driving predictions for the composite primary outcome.

## Results

### Prevalence of Outcome

After data processing, 727,354 pregnancies from Sweden, 5934 pregnancies from Chile, and 37,813 pregnancies from Singapore were used for model development and validation. The final feature sets comprised 33 variables for Sweden, 36 for Chile, and 20 for Singapore (Multimedia Appendix 1). Significant differences were observed across the 3 study populations. Chilean women were younger (mean age 27.56, SD 6.23 y), heavier (mean BMI 28.26, SD 5.62 kg/m^2^), and had higher rates of chronic hypertension (270/5934, 4.55%) and prior stillbirth (97/5934, 1.63%). Swedish women were taller, with lower metabolic risk but a higher prevalence of psychiatric (103,859/727,354, 14.28%) and respiratory conditions (51,358/727,354, 7.06%). Singaporean women were leaner and slightly older, with elevated rates of diabetes mellitus (6142/37,813, 16.24%), reflecting the region’s metabolic risk profile. Regarding lifestyle factors, tobacco use was highest in Chile (461/5934, 7.77%), moderate in Sweden (31,660/727,354, 4.35%), and not available in Singapore. Alcohol consumption was reported in 3.49% (25,385/727,354) of Swedish pregnancies, was nearly absent in Chile, and was not recorded in Singapore.

### Study Outcome

Significant interpopulation differences were observed in both maternal and neonatal outcomes (Table 1). Neonatal morbidity was lowest in Sweden and highest in Chile and Singapore. Preterm birth (<37 wk) occurred in 15.32% (909/5934) of Chilean, 7.74% (2925/37,813) of Singaporean, and only 5.89% (42,805/727,354) of Swedish pregnancies, while low birth weight (<2500 g) was approximately twice as frequent in Chile (551/5934, 9.29%) and Singapore (3584/37,813, 9.48%) as in Sweden (30,308/727,354, 4.17%). Admission to a neonatal intensive care unit for mechanical ventilation or hypoxic-ischemic encephalopathy was markedly more common in Singapore (2658/37,813, 7.03%) and Chile (224/5934, 3.77%) than in Sweden (793/727,354, 0.11%). Maternal morbidity showed a different pattern. Postpartum hemorrhage (>1500 mL) was most frequent in Sweden (22,596/727,354, 3.11%) but less common in Chile (89/5934, 1.50%) and rare in Singapore (86/37,813, 0.23%). Conversely, placental abruption (47/5934, 0.79%) and eclampsia (6/5934, 0.10%) were more prevalent in Chile, while embolism was higher in Singapore (116/37,813, 0.31%) compared with Sweden (328/727,354, 0.05%) and Chile (6/5934, 0.10%). SMM requiring high-dependency or intensive care occurred in 0.24% (14/5934) of Chilean and 0.08% (31/37,813) of Singaporean pregnancies. Maternal deaths were extremely rare across all cohorts (<0.02%). The proportion of pregnancies experiencing the composite primary adverse outcome varied substantially across populations: 10.40% (75,647/727,354) in Sweden, 21.94% (1302/5934) in Chile, and 16.25% (6145/37,813) in Singapore.

**Table 1.** Definition of poor neonatal and maternal outcomes^a^.

Feature	Threshold	Prevalence in Sweden (N=727,354), n (%)	Prevalence in Chile (N=5934), n (%)	Prevalence in Singapore (N=37,813), n (%)	*P* value^b^
Neonatal morbidity					
Gestational age at delivery [23]	<37 wk	42,805 (5.89)	909 (15.32)	2925 (7.74)	<.001
Birth weight (g)	<2500	30,308 (4.17)	551 (9.29)	3584 (9.48)	<.001
Stillbirth	Yes	2542 (0.35)	58 (0.98)	111 (0.29)	<.001
Apgar at 5 min	≤4	4951 (0.68)	2 (0.03)	49 (0.13)	<.001
NICU^c^ (mechanical ventilation or HIE^d^)	Yes	793 (0.11)	224 (3.77)	2658 (7.03)	<.001
Severe maternal morbidity					
Total bleeding (mL)	≥1500	22,596 (3.11)	89 (1.50)	86 (0.23)	<.001
Eclampsia	Yes	225 (0.03)	6 (0.10)	17 (0.04)	.004^e^
Placental abruption	Yes	184 (0.03)	47 (0.79)	166 (0.44)	<.001
Uterine rupture	Yes	633 (0.09)	5 (0.08)	16 (0.04)	.01^e^
Embolism in pregnancy	Yes	328 (0.05)	6 (0.10)	116 (0.31)	<.001^e^
Death from any obstetric cause occurring more than 42 d but <1 y after delivery	Yes	1 (0.00)	1 (0.02)	5 (0.01)	<.001^e^
Admission to SHD^f^ or ICU^g^	Yes	—^h^	14 (0.24)	31 (0.08)	—^i^

a If any of the following conditions are met, the record is labeled “poor neonatal or maternal outcome.”

b *P* values were computed using the Pearson chi-square test on the 3 × 2 contingency table (cohort × event).

c NICU: neonatal intensive care unit.

d HIE: hypoxic ischemic encephalopathy.

e At least one expected cell count under the null hypothesis was <10; therefore, these chi-square *P* values are shown for descriptive comparison only.

f SHD: surgical high dependency.

g ICU: intensive care unit.

h Not applicable.

i Admission to SHD or ICU was not harmonized into the Sweden dataset; therefore, a 3-group test was not possible.

### ML Model Performance

Model performance, as measured by the AUROC, varied across populations and modeling approaches (Table 2). In the Swedish dataset, AUROC values ranged from 0.60 for the base pretrained TabPFN model to 0.65 for both the traditional ensemble models and the TabPFN embedding approach, indicating modest gains with model fine-tuning and feature enrichment. In the Chilean dataset, performance was generally higher, with AUROC values of approximately 0.65 across all approaches, suggesting stable predictive capability even in a smaller but clinically diverse cohort. In contrast, the Singaporean models achieved lower discrimination, with AUROCs of 0.57 to 0.60, reflecting the smaller feature set and more homogenous characteristics. The full results of all ML models trained and tested for all 3 datasets are available in Multimedia Appendix 2. When we looked at the performance metrics for maternal and neonatal outcomes separately, the overall composite outcome seemed to be driven largely by neonatal outcomes in the Sweden dataset, but not in Chile or Singapore (Table 3). This is possibly due to the relatively smaller sample sizes in the latter. LightGBM was chosen as the best-performing model for Sweden, Chile, and Singapore.

**Table 2.** Training and test set splitting and model performance of Tabular Prior-Data Fitted Network and traditional machine learning model.

Risk status	Sweden	Chile	Singapore
Training set, n			
High risk	60,518	1042	4916
Low risk	521,365	3705	25,334
Total	581,883	4747	30,250
Test set, n			
High risk	15,129	260	1229
Low risk	130,342	927	6334
Total	145,471	1187	7563
AI model performance (AUC^a^)			
Base TabPFN^b^	0.60	0.65	0.57
Fine-tuned TabPFN with Sweden	0.63	0.65	0.60
TabPFN embedding (traditional model)	0.65	0.65	0.60
Traditional model	0.65	0.65	0.60
Performance metrics at a fixed false-positive rate of 50%, %			
Sensitivity	69.17	69.62	64.93
Specificity	50.00	50.70	50.06
PPV^c^	13.84	28.37	20.13
NPV^d^	93.32	85.61	88.02

a AUC: area under the curve.

b TabPFN: Tabular Prior-Data Fitted Network.

c PPV: positive predictive value

d NPV: negative predictive value.

**Table 3.** Model discrimination analysis for composite maternal outcomes only and composite neonatal outcomes only.

Outcomes	Sweden	Chile	Singapore
Composite	0.65	0.65	0.60
Neonatal only	0.64	0.65	0.60
Maternal only	0.61	N/A^a^	0.64
Neonatal composite outcome incidence, %	7.58	18.70	15.87
Maternal composite outcome incidence, %	3.22	4.38	0.89

a A maternal-only model is not reported for the Chile cohort because the limited number of positive maternal cases resulted in an unstable model that did not yield a reliable, reportable result.

**Figure 2.**
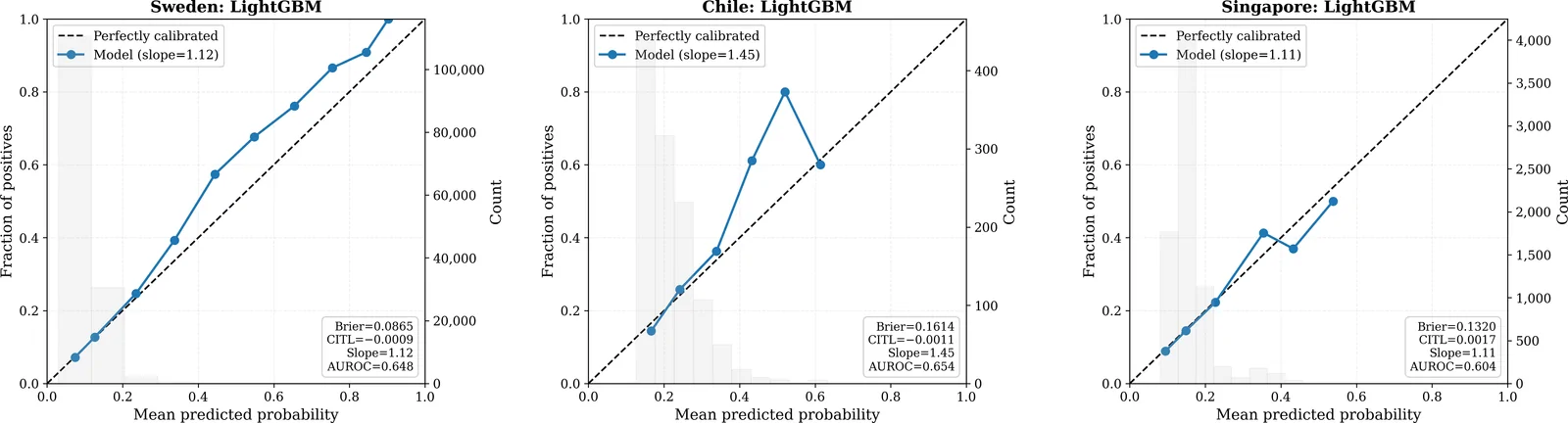
Calibration plots for Sweden, Chile, and Singapore machine learning models. AUROC: area under the receiver operating characteristic; CITL: calibration in the large; LightGBM: light gradient boosting machine.

Across Sweden, Chile, and Singapore, the model calibration plots showed reasonable calibration in Sweden and Singapore, but poorer calibration in Chile, where the calibration slope was approximately 1.45, indicating underconfidence despite acceptable calibration in the large (Figure 2). These findings suggest that the model should be interpreted primarily as a risk-stratification tool rather than as a fully calibrated probability estimator across all cohorts.

At a FPR of 50.00%, the sensitivities for predicting the primary composite adverse outcome were 69.17%, 69.62%, and 64.93% for the Sweden, Chile, and Singapore models, respectively. At a 30.00% FPR operating point, the sensitivities were lower, at 50.38%, 55.38%, and 44.75%, respectively, but with higher PPVs of 16.31%, 34.53%, and 22.32% (Table 4).

**Table 4.** Sensitivity–positive predictive value trade-off at different thresholds.

Metric (%)	Sweden		Chile		Singapore	
	50.00% FPR^a^	30.00% FPR	50.00% FPR	30.00% FPR	50.00% FPR	30.00% FPR
Sensitivity	69.17	50.38	69.62	55.38	64.93	44.75
Specificity	50.00	69.99	50.70	70.55	50.06	69.78
PPV	13.84	16.31	28.37	34.53	20.13	22.32
NPV^b^	93.32	92.40	85.61	84.94	88.02	86.68

a FPR: false-positive rate.

b NPV: negative predictive value.

The sensitivity analysis removing maternal critical care from the Chile dataset showed negligible change in the AUROC of approximately 0.01, reflecting that maternal critical care did not drive prediction in that cohort (Multimedia Appendix 3).

There was minimal temporal drift in model performance, with no appreciable impact of the COVID-19 pandemic when performance was examined across time epochs (Multimedia Appendix 4).

**Figure 3.**
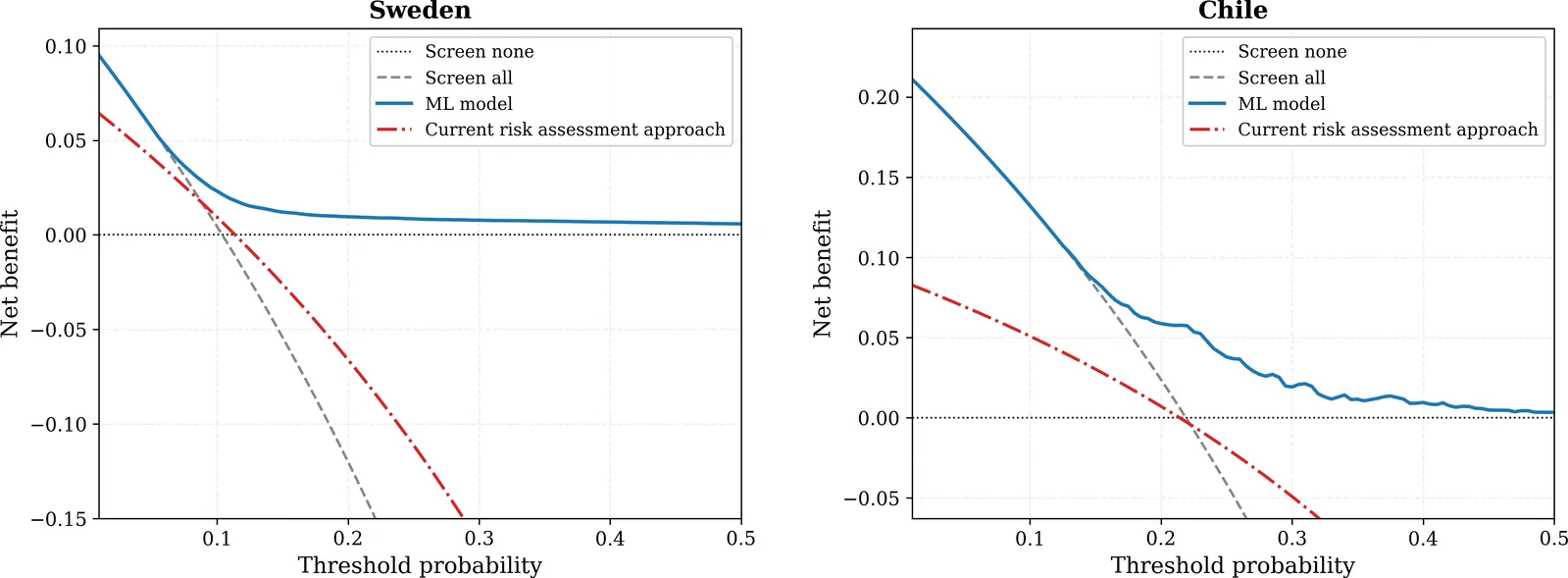
Decision curve analysis plots for Sweden and Chile cohorts assessing net benefit of machine learning models against “screen all,” “screen none,” and current risk assessment strategies. ML: machine learning.

DCA was performed for both the Sweden and Chile cohorts, comparing the net benefit of the ML model against the “screen all” and “screen none” strategies, as well as against the corresponding current clinical risk assessment approach, across threshold probabilities from 1% to 50%. In both cohorts, the ML models demonstrated positive net benefit above the current clinical approach and the “screen all” strategy across clinically plausible thresholds. DCA was not performed for the Singapore cohort because the clinical comparator was a separate prospective birth cohort, precluding a direct comparison within the same population. DCA plots and net benefit values are presented in Figure 3.

### Comparison With Existing Antenatal Risk Assessment Methods

**Figure 4.**
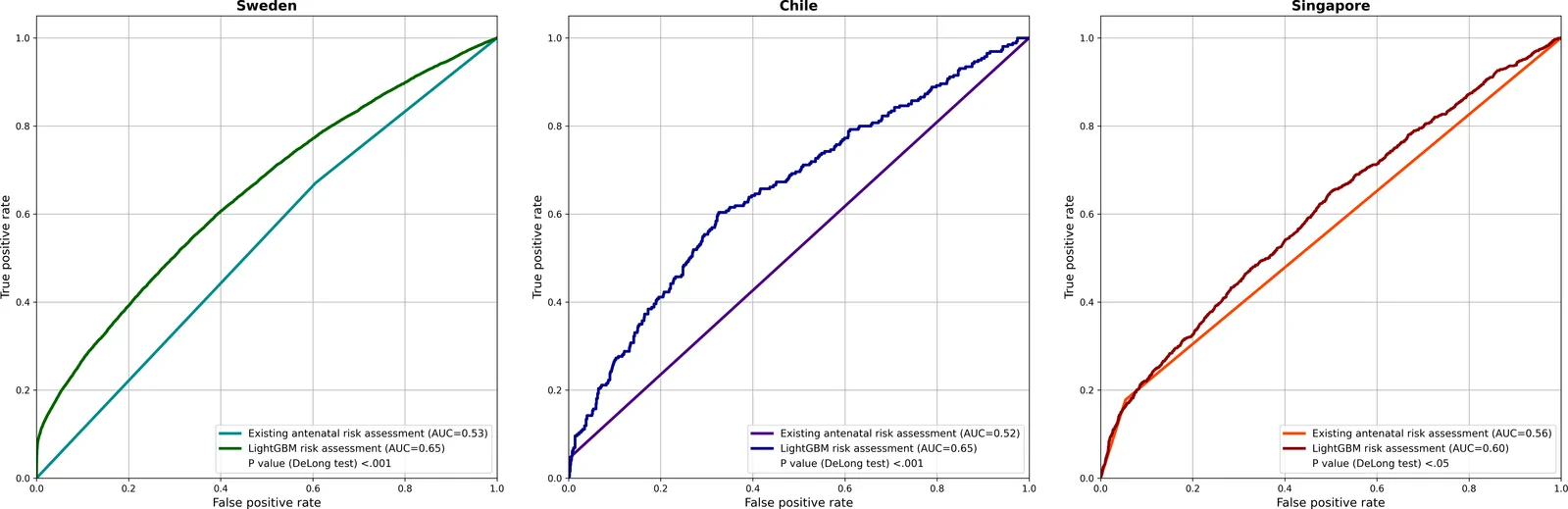
Area under the receiver operating characteristic curves comparing each population’s existing health care professionals’ risk stratification approaches and corresponding top-performing machine learning model. AUC: area under the curve; LightGBM: light gradient boosting machine.

In each population, there were statistically significant differences between the performance of the ML-based risk categorization models and the corresponding real-world clinical risk assessment strategies for identifying pregnancies with the composite adverse outcome. Across all 3 settings, the ML models generally demonstrated improved discrimination compared with conventional approaches (Figure 4). In Sweden, the guideline-based risk assessment achieved an AUROC of 0.53, compared with 0.65 for the traditional ML LightGBM model (*P<*.001). In the Chilean cohort, the midwifery-led risk assessment yielded an AUROC of 0.52, significantly lower than the 0.65 achieved by the traditional ML LightGBM model (*P<*.001). In Singapore, the health care professional–based risk assessment achieved an AUROC of 0.56, significantly lower than the LightGBM model’s 0.60 (*P*<.05).

### Feature Importance Analysis

**Figure 5.**
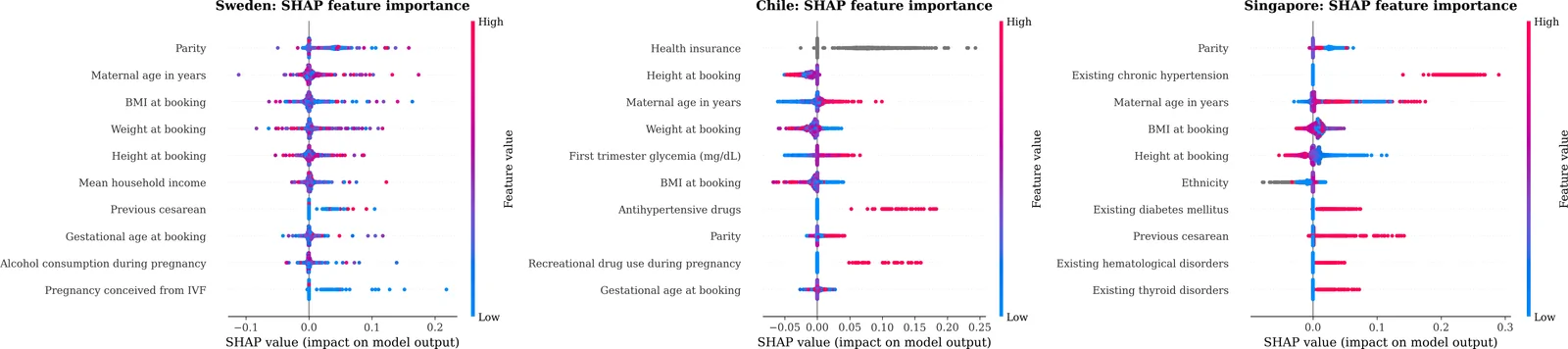
Shapley additive explanations (SHAP) beeswarm plots showing how high and low feature values affect predictions. IVF: in vitro fertilization.

The top-ranked features differed across settings. In the Swedish and Singapore models, sociodemographic characteristics such as maternal age, BMI, parity, ethnicity, and mean household income emerged as the dominant features contributing to the prediction of adverse outcomes. In contrast, the Chilean model was influenced somewhat more by medical and obstetric variables, including antihypertensive use and first-trimester glycemia (Figure 5).

## Discussion

### Main Findings

First, we demonstrated the feasibility of developing and internally validating ML models to identify pregnancies at risk of adverse maternal and neonatal outcomes, individually or in combination, using information routinely collected in the first trimester of pregnancy (<14 weeks’ gestation) across three geographically and socioethnically distinct populations. The cross-population contrasts in maternal characteristics and comorbidity profiles underscore the heterogeneity in risk factors and health care contexts. Sweden represented a high-resource, low-morbidity population with comprehensive health surveillance; Chile exhibited a younger, metabolically at-risk population with higher rates of preterm birth and obstetric complications; while Singapore comprised an older maternity population with a strong metabolic risk profile. These contextual differences reinforce the need for population-specific AI-based antenatal risk assessment models, rather than a single global model trained on heterogeneous data.

Second, from a technical perspective, we employed the recently developed TabPFN architecture, a transformer-based model optimized for tabular data [22]. Despite the methodological rigor and the use of fine-tuning, performance gains were modest. Across all 3 populations, whether using fine-tuned TabPFN, TabPFN-derived embeddings, or traditional ensemble methods, the AUROCs achieved were broadly similar, clustering around 0.60 to 0.65. This similar performance suggests that for first-trimester risk prediction using historical and anthropometric features, the choice of ML algorithm is less critical than the inherent predictive signal in the available features. Traditional ensemble methods may be preferable for clinical deployment given their computational efficiency and established interpretability frameworks. Nonetheless, given that the input features for all models were limited to historical, nonbiological first-trimester measurements (eg, placental biomarkers, serology results, and sonographic markers), this represents a reasonable and pragmatic baseline for early-pregnancy risk stratification. For context, a recent systematic review reported median AUROCs of 0.75 for preeclampsia and 0.65 for spontaneous preterm birth using models that incorporated biomarkers and sonographic data [24]. These findings suggest that our models, based solely on historical and anthropometric inputs, are likely approaching their maximal discriminatory capacity within this limited feature space.

At clinically relevant operating points, the models demonstrated an explicit trade-off between sensitivity and PPV. At a 30.00% FPR, sensitivities of 44.75% to 55.38% were achieved with higher PPVs of 16.31% to 34.53% compared with the metrics at a FPR of 50.00%. This may be preferable in resource-constrained settings where minimizing unnecessary follow-up or referral to specialized services is important. While the discrimination metrics alone may result in hesitancy in clinical deployment, it must be emphasized that these ML models are intended as population-level screening tools rather than diagnostic tests. Moreover, the observed PPVs are comparable to, or if not exceeding, those of widely implemented obstetric screening programs, including combined first-trimester screening for aneuploidy and first-trimester screening for preeclampsia [25,26]. DCA further supported the clinical use of the models, demonstrating positive net benefit above both the current clinical risk assessment approaches and the “screen all” strategy across clinically plausible threshold probabilities in the Sweden and Chile cohorts. These results should be interpreted with the caveat that the clinical comparators are heterogeneous retrospective proxies rather than standardized risk tools.

Third, the comparatively lower discrimination observed in the Singapore models warrants further consideration. While the reduced number of available input variables likely contributed to this finding, several additional factors may also account for the observed performance difference. First, the Singapore dataset was derived from a single tertiary center, resulting in a relatively clinically homogeneous population. Such reduced heterogeneity in both predictors and outcomes may constrain the model’s ability to effectively distinguish between high- and low-risk pregnancies. Second, in comparison with the Swedish and Chilean datasets, several key sociodemographic and behavioral variables were not available, thereby limiting the model’s capacity to incorporate broader determinants of risk that may enhance predictive performance. Third, differences in case mix and outcome distribution, including a comparatively narrower spectrum of disease severity, may have further attenuated model discrimination. As model performance is inherently contingent upon the informational richness and heterogeneity of the training data, these findings suggest that the predictive ceiling of first-trimester models based on limited, nonbiological features may vary across health care settings and data environments. An additional unexpected finding in the Singapore cohort was the model’s better discrimination for maternal outcomes alone (AUROC 0.64) compared to the overall composite (0.60) and neonatal outcomes (AUROC 0.60). Given the low incidence of maternal morbidity in the Singapore cohort (0.89%), this finding may reflect overfitting to a handful of severe maternal cases rather than generalizable risk patterns and therefore reduces model stability. This highlights the importance of considering sample size when interpreting subgroup analyses for rare outcomes.

Finally, the SHAP analysis offered important insights into model interpretability and context-specific drivers of prediction. In both Sweden and Singapore, sociodemographic factors emerged as the most influential predictors of adverse outcomes, whereas in Chile, obstetrical and clinical factors predominated. It is essential to clarify that SHAP “importance” reflects a feature’s contribution to model discrimination, not its prevalence or causal strength. Features with high SHAP values are those the model most often uses to differentiate between high- and low-risk pregnancies, not necessarily those most prevalent or severe in the population. In addition, SHAP analysis reflects the availability of features in each specific dataset rather than inherent differences in biological or social risk drivers. Nevertheless, the prominence of sociodemographic variables’ contribution in Sweden and Singapore suggests that these nonclinical attributes may capture cumulative social disadvantage, access inequities, or latent vulnerability that traditional clinical risk models often overlook. This aligns with growing evidence that social determinants of health (SDH), including socioeconomic stability, educational attainment, and access to care, profoundly shape perinatal outcomes even in high-resource settings [27]. Properly interpreted, AI-based models can serve as a tool to uncover hidden social gradients in pregnancy health.

### Comparison With Other Studies

Although several risk prediction tools exist for specific conditions such as preeclampsia, stillbirth, and preterm birth, few examples exist of ML being applied to comprehensive antenatal risk assessment [28-31]. Escobar et al [32] used over 300,000 electronic health records from Kaiser Permanente Northern California to develop ML models predicting adverse obstetric and neonatal complications, achieving an area under the curve of 0.79. However, these models relied primarily on intrapartum data including vital signs, labor progress parameters, and laboratory results. In contrast, our models rely solely on first-trimester information, offering the potential to stratify risk and guide early interventions. Similarly, Pan et al [33] applied ML to a smaller cohort (6457 women of low socioeconomic status) to predict adverse birth outcomes for social service allocation, reporting up to a 36% improvement over paper-based risk assessments. Together with these studies, our findings highlight the potential of using readily available demographic and early pregnancy data to enable scalable and equitable antenatal risk assessment, facilitating targeted support and early preventive strategies for pregnant women most in need.

### Strengths and Limitations

A key strength of this study is in its use of large, high-quality datasets encompassing over half a million pregnancies, from diverse socioeconomic and health care settings. The inclusion of variables routinely available at the first antenatal contact enhances generalizability and supports real-world implementation. By comparing three distinct health settings, we also demonstrate the adaptability of a single AI pipeline across varied data environments.

Notwithstanding the strengths of this study, several limitations should be acknowledged in the interest of transparency and balanced interpretation. First, as with all retrospective ML studies, inherent limitations related to the underlying data sources must be recognized. Although differences in variable definitions, data completeness, and health care delivery models were generally mild, they may nonetheless have introduced residual bias and influenced model performance. Socioeconomic and behavioral data were more comprehensively captured in Sweden than in Chile or Singapore. Information on behavioral risks (eg, tobacco use and alcohol consumption in pregnancy) was unavailable in some datasets, potentially underestimating the influence of modifiable lifestyle factors. Future models integrating biomarker, imaging, and environmental data collected at designated pregnancy time points, together with standardized social metrics may substantially enhance predictive accuracy.

Second, the use of proxy variables for current clinical risk assessment across the three settings may not fully capture the granularity or nuances of real-time clinical evaluation. In the Sweden cohort, the approach used reflects adherence to guidelines rather than real-time clinical judgment. In the Chile and Singapore cohorts, referral to high-risk services was used but the disadvantage is that system-level factors including referral thresholds and service availability could have influenced the referral decisions and therefore not purely reflected clinicians’ risk categorization. The heterogeneous operationalization of clinical practice across the various settings of this study reflects real-world data constraints and underscores the need for prospective studies directly measuring clinician risk perception, a gap that only randomized controlled trials will be able to address. To this end, the principal investigators have initiated a randomized controlled trial evaluating the impact of AI-assisted risk stratification versus standard care on pregnancy outcomes within an intention-to-treat framework (ClinicalTrials.gov identifier: NCT06974188).

Third, a limitation in using simple mean and mode imputation is that it may underestimate variance compared to multiple imputation approaches. This approach was selected primarily because missingness was low within each country-specific dataset, and the primary objective was to support robust model training using tree-based ensemble methods, which are generally tolerant to moderate imputation error, rather than to derive unbiased parameter estimates. Given the large overall sample size, particularly in the Swedish cohort, and the low-to-moderate level of missingness, any impact on model discrimination is likely to be limited, especially in the context of predictive performance rather than statistical inference. Although multiple imputation may offer advantages in terms of statistical efficiency in certain settings, it introduces additional assumptions regarding missingness mechanisms and substantially increases computational complexity. In particular, its integration within cross-validation and ensembling pipelines across heterogeneous datasets with differing structures and variable definitions would likely confer only marginal gains in predictive performance for tree-based models.

Fourth, although the primary objective of the models was risk stratification rather than direct probability estimation, calibration remains relevant when predicted risks are interpreted clinically. The weaker calibration observed in Chile, reflected by a calibration slope of approximately 1.45, indicates that predicted probabilities in this cohort should be interpreted cautiously and may require recalibration before clinical deployment.

Lastly, we acknowledge that the comparison of retrospectively developed predictive models against real-world clinical practice subjects the findings to the “treatment paradox” or intervention bias. The identification of high-risk pregnancies may have resulted in interventions that averted the adverse outcome and therefore led to the conversion of “true positives” in the clinical baseline to “false positives” in the retrospective analyses. The magnitude of incremental benefit shown in the DCA may therefore overestimate the true added value in prospective development. Prospective validation through randomized controlled trials is essential to accurately quantify the true clinical utility of AI-assisted risk stratification. From this perspective, the observed performance of the ML models reflects their ability to identify patients who remain at elevated risk despite current risk assessment approaches being used. Therefore, the ML models presented in this study should be interpreted as adjunct tools with the potential to predict residual risk under real-world care pathways, rather than tools used to replace clinical judgment altogether.

### Potential Clinical Applications

There is potential value of integrating such AI tools into routine antenatal care, especially as a decision-support tool. When adopted within a graduated antenatal care framework, it should raise clinical awareness, rather than immediate referral to specialized services for high-risk pregnancies, or a binary treat-or-no-treat paradigm. Potential applications include prefirst visit self-assessment via a patient-facing application, enabling preliminary risk estimation and immediate triage into appropriate low- or high-risk care pathways even at the initial antenatal encounter. Importantly, these models are intended not to replace clinical judgment but to augment clinician decision-making through a hybrid AI-human approach, with the potential to complement existing standards of care. Finally, our work aligns with the broader vision of Universal Maternal AI—a holistic, equitable approach to pregnancy health that integrates social, demographic, and behavioral determinants beyond traditional biomedical models. By framing pregnancy as a continuum of well-being rather than a binary state of disease versus normalcy, such AI frameworks, augmented by clinical insight, hold promise for guiding personalized, preventive, and socially attuned maternity care globally.

### Conclusion

In this multicenter study, we developed and internally validated first-trimester ML-based antenatal risk assessment models across three geographically and socioethnically distinct populations and observed that these models generally showed improved performance compared with existing first-trimester clinical risk stratification approaches. Using routinely collected early pregnancy data, the models achieved moderate discrimination and demonstrated stable performance over time, with sociodemographic variables contributing meaningfully to risk differentiation. Taken together, our findings suggest the potential feasibility of population-specific, AI-based risk stratification as an adjunct to current antenatal care, particularly as a clinical decision-support tool to enhance early pregnancy risk awareness within established antenatal care frameworks.

## Supplementary material

10.2196/88450 Multimedia Appendix 1 Demographics and clinical characteristics of patients from Sweden, Chile, and Singapore datasets.

10.2196/88450 Multimedia Appendix 2 Details of model performance.

10.2196/88450 Multimedia Appendix 3 Sensitivity analysis removing maternal critical care from the Chile dataset.

10.2196/88450 Multimedia Appendix 4 Examination of the extent of temporal drift due to the COVID-19 pandemic on the machine learning model from Singapore.

## Abbreviations

AUROC: area under the receiver operating characteristic
DCA: decision curve analysis
FPR: false-positive rate
LightGBM: light gradient boosting machine
ML: machine learning
PPV: positive predictive value
SHAP: Shapley additive explanations
SMM: severe maternal morbidity
TabPFN: Tabular Prior-Data Fitted Network

## References

[1] (2023). Trends in maternal mortality 2000 to 2020: estimates by WHO, UNICEF, UNFPA, World Bank Group and UNDESA/population division. https://iris.who.int/server/api/core/bitstreams/c3957b94-cdd5-47d7-85f8-6202be229f8e/content.

[2] Howell EA (2018). Reducing disparities in severe maternal morbidity and mortality. Clin Obstet Gynecol.

[3] Geller SE, Koch AR, Garland CE, MacDonald EJ, Storey F, Lawton B (2018). A global view of severe maternal morbidity: moving beyond maternal mortality. Reprod Health.

[4] Grobman WA, Bailit JL, Rice MM (2014). Frequency of and factors associated with severe maternal morbidity. Obstet Gynecol.

[5] Say L, Chou D, Gemmill A (2014). Global causes of maternal death: a WHO systematic analysis. Lancet Glob Health.

[6] Callaghan WM, Grobman WA, Kilpatrick SJ, Main EK, D’Alton M (2014). Facility-based identification of women with severe maternal morbidity: it is time to start. Obstet Gynecol.

[7] Knight M, Lewis G, Acosta CD, Kurinczuk JJ (2014). Maternal near-miss case reviews: the UK approach. BJOG.

[8] Lawton B, MacDonald EJ, Brown SA (2014). Preventability of severe acute maternal morbidity. Am J Obstet Gynecol.

[9] Vause S, Clarke B (2014). Risk stratification and hierarchy of antenatal care. Best Pract Res Clin Obstet Gynaecol.

[10] Clarke E, Cade TJ, Brennecke S (2020). Early pregnancy screening for women at high-risk of GDM results in reduced neonatal morbidity and similar maternal outcomes to routine screening. J Pregnancy.

[11] Ng YHG, Wright A, Ng YYV (2025). High-risk consult multidisciplinary team in a tertiary maternity unit: changing prevalence of cases. J Multidiscip Healthc.

[12] Berglund A, Lindberg M, Nyström L, Lindmark G (2007). Combining the perspectives of midwives and doctors improves risk assessment in early pregnancy. Acta Obstet Gynecol Scand.

[13] Braveman PA, Heck K, Egerter S (2015). The role of socioeconomic factors in Black-White disparities in preterm birth. Am J Public Health.

[14] Panch T, Szolovits P, Atun R (2018). Artificial intelligence, machine learning and health systems. J Glob Health.

[15] Kim HY, Cho GJ, Kwon HS (2023). Applications of artificial intelligence in obstetrics. Ultrasonography.

[16] Berghella V, Palacio M, Ness A, Alfirevic Z, Nicolaides KH, Saccone G (2017). Cervical length screening for prevention of preterm birth in singleton pregnancy with threatened preterm labor: systematic review and meta-analysis of randomized controlled trials using individual patient-level data. Ultrasound Obstet Gynecol.

[17] Khalifeh A, Quist-Nelson J, Berghella V (2017). Universal cervical length screening for preterm birth prevention in the United States. J Matern Fetal Neonatal Med.

[18] Watson HA, Carlisle N, Seed PT (2021). Evaluating the use of the QUiPP app and its impact on the management of threatened preterm labour: a cluster randomised trial. PLoS Med.

[19] Sarno L, Neola D, Carbone L (2023). Use of artificial intelligence in obstetrics: not quite ready for prime time. Am J Obstet Gynecol MFM.

[20] Graviditetsregistret [Article in Swedish].

[21] Statistikmyndigheten SCB [Article in Swedish].

[22] Hollmann N, Müller S, Purucker L (2025). Accurate predictions on small data with a tabular foundation model. Nature.

[23] American College of Obstetricians and Gynecologists Preterm labor and birth. Every Stage Health.

[24] van Eekhout JCA, Becking EC, Scheffer PG (2025). First-trimester prediction models based on maternal characteristics for adverse pregnancy outcomes: a systematic review and meta-analysis. BJOG.

[25] Li SW, Barrett AN, Gole L (2015). The assessment of combined first trimester screening in women of advanced maternal age in an Asian cohort. Singapore Med J.

[26] Tan MY, Wright D, Syngelaki A (2018). Comparison of diagnostic accuracy of early screening for pre-eclampsia by NICE guidelines and a method combining maternal factors and biomarkers: results of SPREE. Ultrasound Obstet Gynecol.

[27] He Z, Pfaff E, Guo SJ (2023). Enriching real-world data with social determinants of health for health outcomes and health equity: successes, challenges, and opportunities. Yearb Med Inform.

[28] Jhee JH, Lee S, Park Y (2019). Prediction model development of late-onset preeclampsia using machine learning-based methods. PLoS One.

[29] Malacova E, Tippaya S, Bailey HD (2020). Stillbirth risk prediction using machine learning for a large cohort of births from Western Australia, 1980-2015. Sci Rep.

[30] Montgomery-Csobán T, Kavanagh K, Murray P (2024). Machine learning-enabled maternal risk assessment for women with pre-eclampsia (the PIERS-ML model): a modelling study. Lancet Digit Health.

[31] Weber A, Darmstadt GL, Gruber S (2018). Application of machine-learning to predict early spontaneous preterm birth among nulliparous non-Hispanic Black and White women. Ann Epidemiol.

[32] Escobar GJ, Soltesz L, Schuler A, Niki H, Malenica I, Lee C (2021). Prediction of obstetrical and fetal complications using automated electronic health record data. Am J Obstet Gynecol.

[33] Pan I, Nolan LB, Brown RR (2017). Machine learning for social services: a study of prenatal case management in Illinois. Am J Public Health.

