# Supplementary material for: Machine Learning–Based First-Trimester Antenatal Risk Prediction for Adverse Maternal and Neonatal Outcomes: Multicenter Model Development Study
Source: J Med Internet Res. 2026 Aug 26;28:e88450. doi: 10.2196/88450 (PMC13519590; doi:10.2196/88450)
Supplement: Supplementary file 1 [file jmir-v28-e88450-s001.docx]

**Supplementary Table 1.** Demographics and clinical characteristics of patients from Sweden^, Chile and Singapore datasets. Data are in percentages (%) or mean ± standard error of the mean. Abbreviations: SEM- standard error of mean, N.A. – Not Available, BMI – body mass index, IVF – in vitro fertilization.

| **Characteristics** | **Swedish**  (n=727,354) | **Chilean**  (n=5,934) | **Singaporean**  (n=37,813) | **P-value*** |
| --- | --- | --- | --- | --- |
| Maternal Age in Years | 31.10±0.01 | 27.56±0.08 | 31.68±0.02 | <.001 |
| Height at Booking | 165.95±0.01 | 159.02±0.00 | 158.40±0.00 | <.001 |
| Weight at Booking | 69.06±0.02 | 71.44±0.20 | N.A. | <.001 |
| BMI at Booking | 25.07±0.01 | 28.26±0.07 | 25.66±0.04 | <.001 |
| Mean Household Income #(Thousand Swedish Krona/year) | 459.68±0.10 | N.A. | N.A. | - |
| Health Insurance | N.A | χ | N.A | - |
| Single- not Married | 2.09%  (15,234/727,354) | N.A. | N.A. | - |
| Cohabiting with Child's Father | 91.73%  (667,200/727,354) | N.A. | N.A. | - |
| Country of birth- Sweden | 73.71%  (536,162/727,354) | N.A. | N.A. | - |
| Chilean National | N.A. | 81.97%  (4,864/5,934) | N.A. | - |
| Existing Chronic Hypertension | 0.50%  (3,640/727,354) | 4.55%  (270/5,934) | 3.81%  (1,442/37,813) | <.001 |
| Antihypertensive Drug Use | N.A. | 3.61% (214/5,934) | N.A. | - |
| Previous Cardio-vascular Disease | 1.47%  (10,660/727,354) | 0.46%  (27/5,934) | 0.54%  (204/37,813) | <.001 |
| Existing Respiratory Disease | 7.06%  (51,358/727,354) | 2.54%  (151/5,934) | 0.54%  (204/37,813) | <.001 |
| Existing Chronic Kidney Disease | 0.45%  (3,239/727,354) | 0.17%  (10/5,934) | 0.26%  (98/37,813) | <.001 |
| Existing Endocrine Disorders | 5.89%  (42,875/727,354) | 0.72%  (43/5,934) | 0.06%  (21/37,813) | <.001 |
| Existing Diabetes Mellitus | 0.90%  (6,513/727,354) | 2.04%  (121/5,934) | 16.24%  (6,142/37,813) | <.001 |
| Existing Insulin Resistance | N.A. | 2.93% (174/5,934) | N.A. | - |
| Existing Autoimmune Disease | 0.16%  (1,157/727,354) | 0.15%  (9/5,934) | 0.34%  (129/37,813) | <.001 |
| Existing Thyroid Disorders | N.A. | 4.11%  (244/5,934) | 1.90%  (719/37,813) | <.001 |
| Existing Neurological Disorders | 0.54%  (3,964/727,354) | 0.71%  (42/5,934) | 0.29%  (108/37,813) | <.001 |
| Existing Psychiatric Disorders | 14.28%  (103,859/727,354) | 2.43%  (144/5,934) | N.A. | <.001 |
| History of Gynecological Disorders | 15.70%  (114,225/727,354) | 4.23%  (251/5,934) | N.A. | <.001 |
| Existing Liver Disease | N.A. | 0.19%  (11/5,934) | 0.56%  (213/37,813) | <.05 |
| Maternal Biliary Disease | N.A. | 1.20% (71/5,934) | N.A. | - |
| History of Jaundice | 0.69%  (5,037/727,354) | N.A. | N.A. | - |
| Existing Bowel Disease | 0.82%  (5,973/727,354) | 0.07%  (4/5,934) | N.A. | <.001 |
| Previous Recurrent Urinary Tract Infections | 10.53%  (76,585/727,354) | 2.33%  (138/5,934) | 0.26%  (98/37,813) | <.001 |
| Existing Hematological Disorders | N.A. | N.A. | 2.36%  (891/37,813) | N.A. |
| History of Vascular Thrombosis | 0.81%  (5,875/727,354) | 0.12%  (7/5,934) | 0.31%  (116/37,813) | <.001 |
| Gestational Age at Booking | 9.07±0.01 | 11.89±0.07 | N.A. | <.001 |
| Parity | 0.89±0.00 | 1.05±0.01 | 0.85±0.01 | <.001 |
| Gravidity | N.A. | 1.28±0.02 | N.A. | - |
| Pregnancy conceived from IVF | 4.35%  (31,635/727,354) | 0.07%  (4/5,934) | N.A. | <.001 |
| IVF in previous pregnancy | 3.87% (28,184/727,354) | N.A. | N.A. | - |
| First Trimester Glycemia (mg/dl) | N.A. | 80.89±0.19 | N.A. | N.A. |
| Number of Previous Termination of Pregnancy (Mean± SEM) | N.A. | 0.23±0.01 | N.A. | N.A. |
| Number of Stillbirths >=1 | 0.78%  (5,671/727,354) | 1.63%  (97/5,934) | 0.81%  (305/37,813) | <.001 |
| History of Pre-Eclampsia | 3.04%  (22,142/727,354) | N.A. | 3.81%  (1,442/37,813) | <.001 |
| History of Eclampsia | 0.03%  (225/727,354) | 0.10%  (6/5,934) | N.A. | <.05 |
| Previous Caesarean | 10.35%  (75,263/727,354) | 18.39%  (1,091/5,934) | 17.58%  (6,648/37,813) | <.001 |
| Snuff§ use at Booking | 1.14%  (8,321/727,354) | N.A. | N.A. | - |
| Tobacco Consumption during Pregnancy | 4.35%  (31,660/727,354) | 7.77%  (461/5,934) | N.A. | <.001 |
| Recreational Drug Use during Pregnancy | N.A. | 2.83%  (168/5,934) | N.A. | - |
| Alcohol Consumption During Pregnancy | 3.49%  (25,385/727,354) | 2.98%  (177/5,934) | N.A. | <.001 |

*Statistical significance is denoted as P <.05 or P <.001.

\# Household income is estimated by average household income per commune from 2011 to 2018.

χ Health insurance was a categorical variable denoted by A,B,C,D.

§**Snuff** is a form of smokeless tobacco, popular in Sweden. It is a moist, finely ground mixture of tobacco, water, and salt, traditionally placed under the upper lip.

^ Swedish education-level and employment-status categories are shown below.

Education level (Graviditetsregistret MHV1) — Swedish cohort, n=727,354

| **Category (English / Swedish)** | **Swedish, n (%)** |
| --- | --- |
| Other education (Annan utbildning) | 39,677 (5.45%) |
| Primary / compulsory school (Folkskola/Grundskola) | 13,092 (1.80%) |
| University / higher education (Universitet eller högskola) | 383,874 (52.78%) |
| Up to and including upper secondary (Upp till och med gymnasium) | 230,213 (31.65%) |
| Don't know / prefer not to say (Vet ej) | 60,498 (8.32%) |

Employment status (Graviditetsregistret MHV1) — Swedish cohort, n=727,354

| **Category (English / Swedish)** | **Swedish, n (%)** |
| --- | --- |
| Other (Annat) | 27,946 (3.84%) |
| Job-seeker / unemployed (Arbetssökande) | 24,352 (3.35%) |
| Gainfully employed (Förvärvsarbetande) | 535,677 (73.65%) |
| On parental leave (Föräldraledig) | 49,041 (6.74%) |
| On sick leave (Sjukskriven) | 12,054 (1.66%) |
| Student (Studerande) | 72,859 (10.02%) |
| Don't know / prefer not to say (Vet ej) | 5,425 (0.75%) |

Health insurance (FONASA group) — Chilean cohort, n=5,934

| **FONASA group** | **Chilean, n (%)** |
| --- | --- |
| A (lowest income) | 2,898 (48.84%) |
| B | 1,602 (27.00%) |
| C | 764 (12.87%) |
| D (highest income) | 649 (10.94%) |
| Other / unspecified | 21 (0.35%) |

Race / ethnicity — Singaporean cohort, n=37,813

| **Race / ethnicity** | **Singaporean, n (%)** |
| --- | --- |
| Chinese | 10,564 (27.94%) |
| Malay | 7,703 (20.37%) |
| Indian | 4,822 (12.75%) |
| Other | 4,977 (13.16%) |
| Not recorded | 9,747 (25.78%) |

Variable notes (model-input variables reported in this table):

- The Chilean "Existing Diabetes Mellitus" value is pre-existing (pregestational) diabetes — the model input; gestational diabetes (a later-pregnancy diagnosis) is excluded.

- "Previous Caesarean" is the proportion of women with at least one previous caesarean delivery (all three cohorts).

- Alcohol consumption in the Swedish cohort is modelled as a continuous AUDIT-points score (mean 1.77 ± 0.002); the percentage shown is a binary alcohol-use indicator. The Chilean value is the binary first-visit alcohol-consumption variable.
