# Supplementary material for: Machine Learning–Based First-Trimester Antenatal Risk Prediction for Adverse Maternal and Neonatal Outcomes: Multicenter Model Development Study
Source: J Med Internet Res. 2026 Aug 26;28:e88450. doi: 10.2196/88450 (PMC13519590; doi:10.2196/88450)
Supplement: Supplementary file 2 [file jmir-v28-e88450-s002.docx]

**Supplementary Table 2.** Details of model performance. Abbreviations: TabPFN - Tabular Prior-data Fitted Network, LightGBM - Light Gradient-Boosting Machine, XGBoost - eXtreme Gradient Boosting

| Model/Method | TabPFN Embedding  (Traditional Model) | | | Traditional Model | | |
| --- | --- | --- | --- | --- | --- | --- |
|  | Sweden | Chile | Singapore | Sweden | Chile | Singapore |
| LightGBM | 0.65 | 0.64 | 0.59 | 0.65 | 0.65 | 0.60 |
| CatBoost | 0.65 | 0.65 | 0.60 | 0.65 | 0.65 | 0.60 |
| RandomForest | 0.63 | 0.63 | 0.55 | 0.64 | 0.62 | 0.55 |
| XGBoost | 0.65 | 0.63 | 0.59 | 0.64 | 0.61 | 0.60 |
| ExtraTrees | 0.64 | 0.63 | 0.55 | 0.62 | 0.60 | 0.54 |
| KNeighbours | 0.55 | 0.51 | 0.53 | 0.55 | 0.54 | 0.52 |
