# Supplementary material for: Machine Learning–Based First-Trimester Antenatal Risk Prediction for Adverse Maternal and Neonatal Outcomes: Multicenter Model Development Study
Source: J Med Internet Res. 2026 Aug 26;28:e88450. doi: 10.2196/88450 (PMC13519590; doi:10.2196/88450)
Supplement: Supplementary file 3 [file jmir-v28-e88450-s003.docx]

**Supplementary Table 3.** Sensitivity analysis removing maternal critical care from the Chile dataset

*Step 1: Determining contribution of maternal critical care to composite outcome*

| Country | Critical Care Contribution (%) | Analysis Needed? |
| --- | --- | --- |
| Singapore | 1.50 | No |
| Chile | 9.09 | Yes |

*Step 2: Sensitivity analysis on Chile dataset.*

| Outcome Definition | AUROC |
| --- | --- |
| With critical care in outcome | 0.65 |
| Without critical care in outcome | 0.66 |
| Difference | - 0.01 |
