# Supplementary material for: Machine Learning–Based First-Trimester Antenatal Risk Prediction for Adverse Maternal and Neonatal Outcomes: Multicenter Model Development Study
Source: J Med Internet Res. 2026 Aug 26;28:e88450. doi: 10.2196/88450 (PMC13519590; doi:10.2196/88450)
Supplement: Supplementary file 4 [file jmir-v28-e88450-s004.docx]

**Supplementary Table 4. Examination of the extent of temporal drift due to the COVID-19 pandemic, on the ML model from Singapore.**

| Approach | Test Set | N (test) | AUROC | Sensitivity at 50% Specificity threshold |
| --- | --- | --- | --- | --- |
| Random 80/20 | Mixed years | 7,563 | 0.60 | 63.63% |
| Temporal | *2020-2022 (aggregate) | 15,327 | 0.60 | 62.01% |
| Temporal | 2020 only | 5,097 | 0.58 | 59.57% |
| Temporal | 2021 only | 5,377 | 0.61 | 62.92% |
| Temporal | 2022 only | 4,853 | 0.60 | 63.24% |

**2020-2022 taken as COVID-19 pandemic years*

*Singapore was selected for the temporal-drift analysis because its dataset spans 2015–2022, allowing the establishment of a clear five-year pre-pandemic training baseline (2015–2019) and three complete, distinct pandemic-era test years (2020, 2021, 2022). In contrast, the Swedish dataset concluded in 2021, with an incomplete 2021 and no 2022 available, limiting its suitability for isolating temporal-drift effects with the pandemic period*. *The Chile dataset was comparatively too small to perform a meaningful analysis.*
